# Supplementary figures and images for: The dynamics of alternative pathways to compensatory substitution
Source: BMC Bioinformatics. 2013 Oct 15;14(Suppl 15):S2. doi: 10.1186/1471-2105-14-S15-S2 (PMC3852009; doi:10.1186/1471-2105-14-S15-S2)

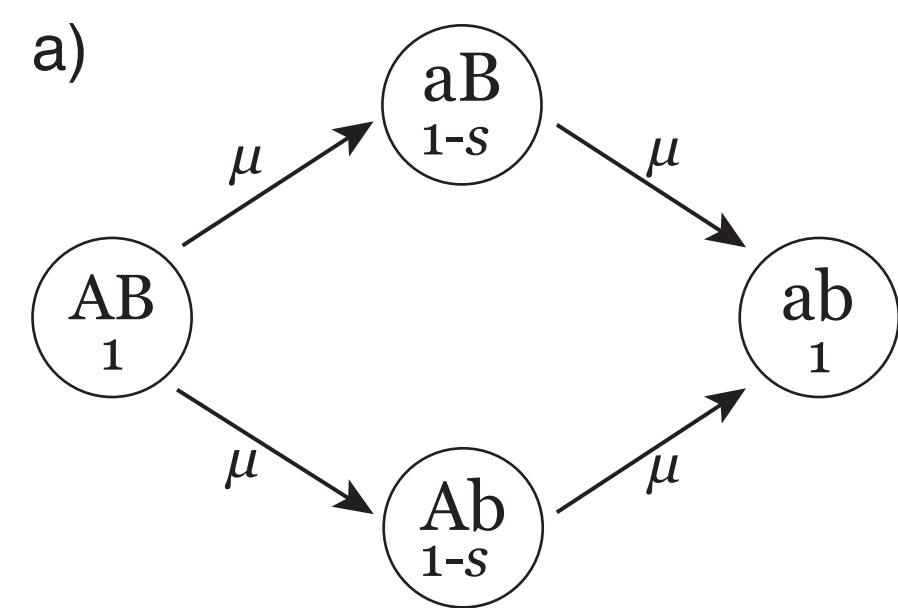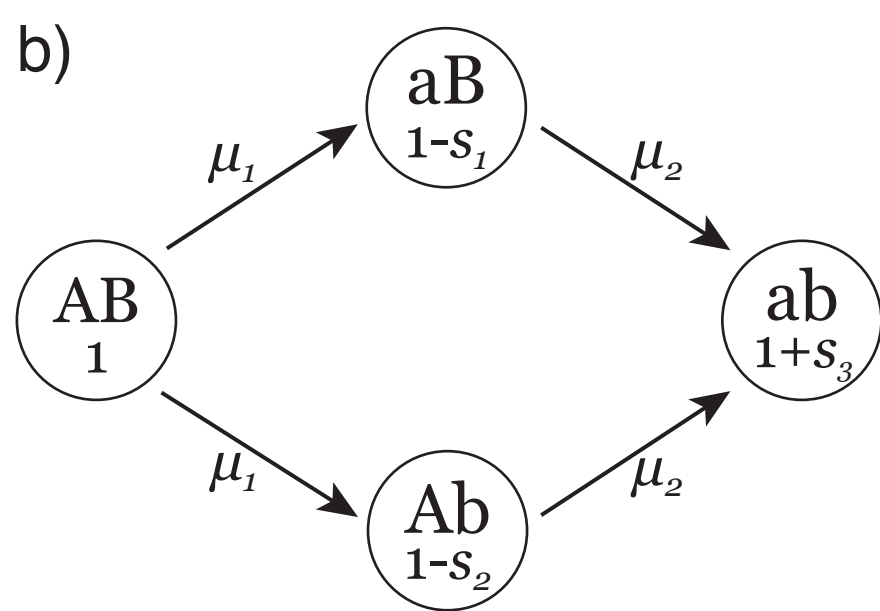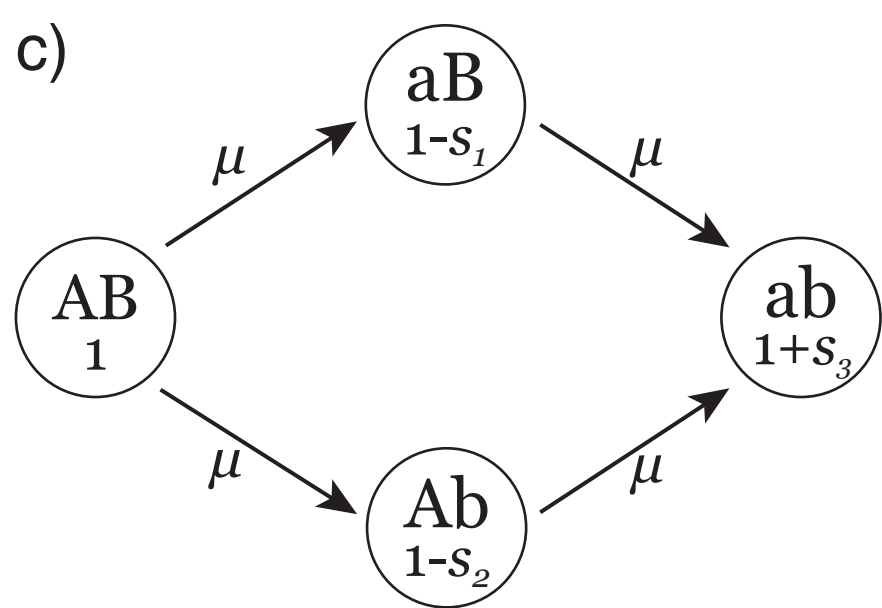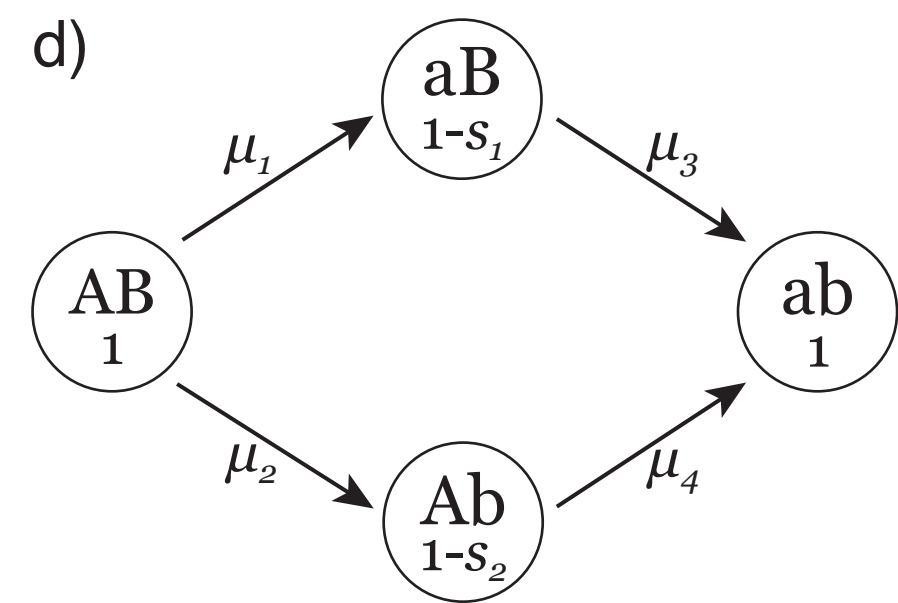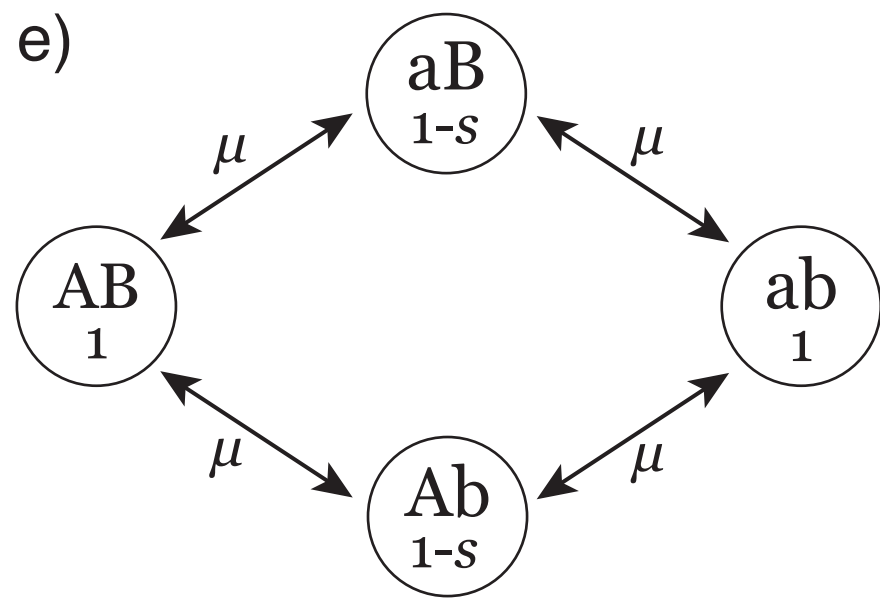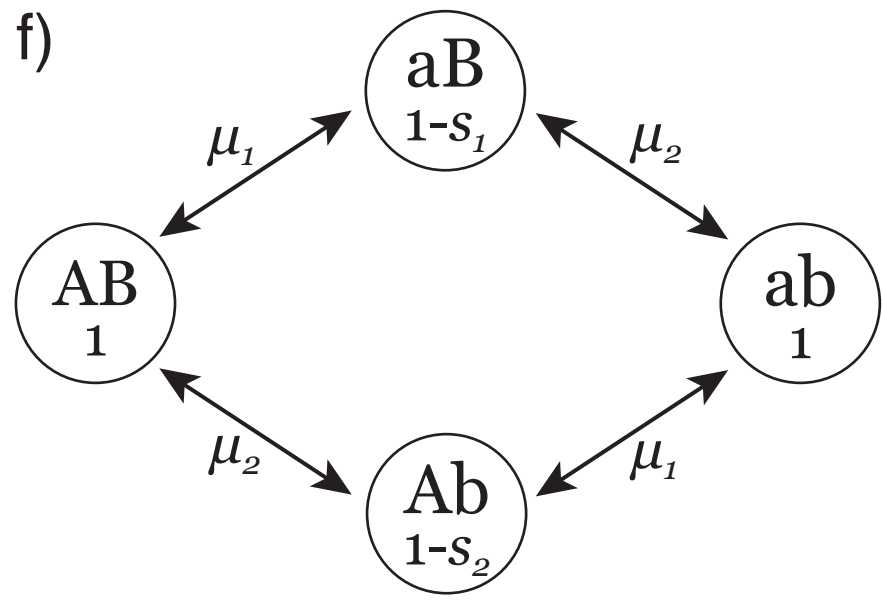

Supplement: Additional file 1 — Mutation-selection models used previously for study of the compensatory substitution process in a population. (a) Kimura (1985) (b) Iizuka and Takefu (1996) (c) Michalakis and Slatkin (1996) (d) Stephan (1996) (e) Higgs (1998) (f) Innan and Stephan (2001) [file 1471-2105-14-S15-S2-S1.PDF]

Returns to AB

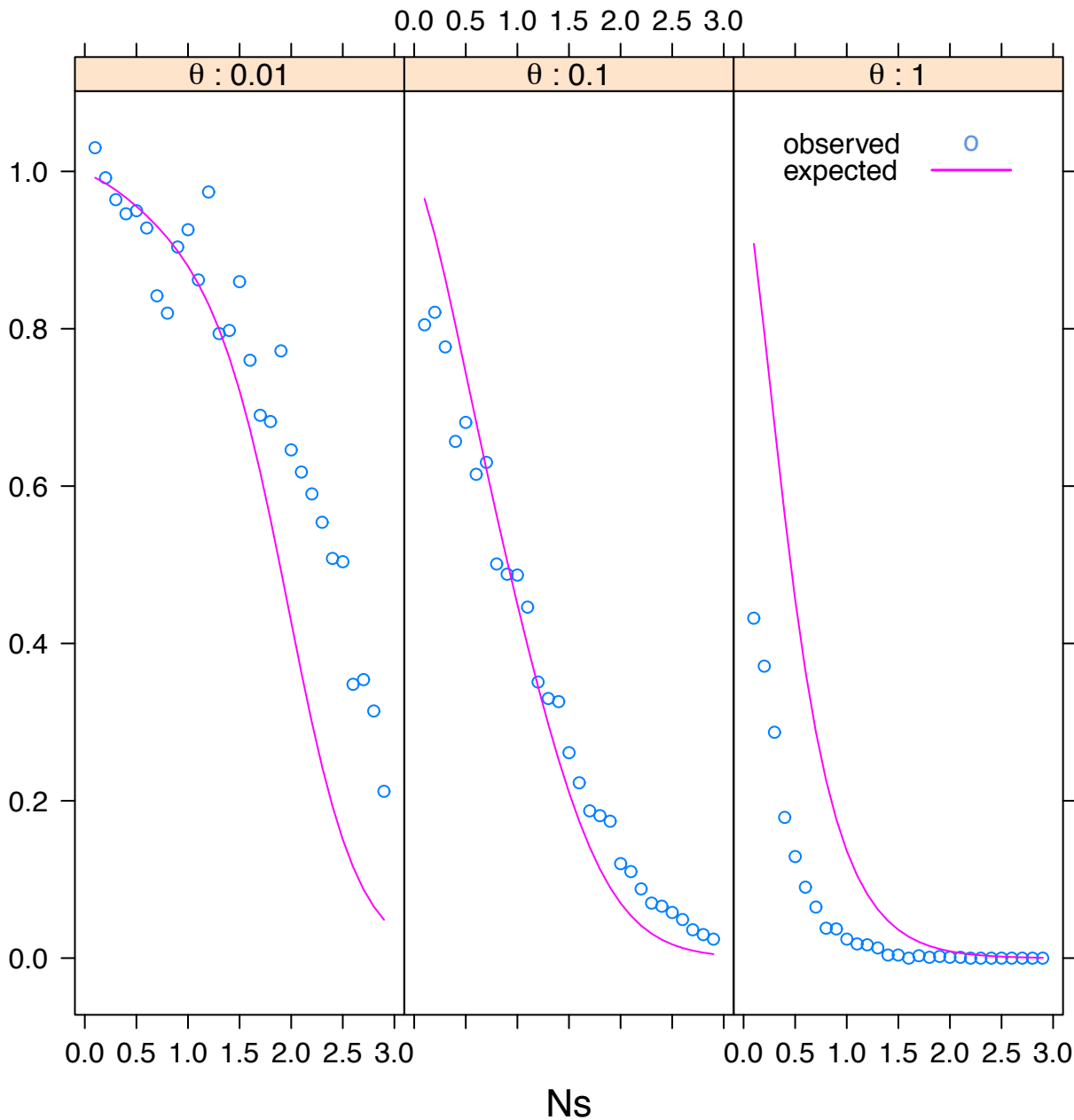

Supplement: Additional file 2 — Reversions to the ancestral state under recombination. The average number of times returning to state AB after having been fixed for a deleterious intermediate state are shown under four mutation rates (panels) across a variety of selection coefficients. The analytical predictions (lines) from β of the number of recursions compared to simulations in the presence of recombination (points). Data for θ = 0.001 is not shown due to low sample size. [file 1471-2105-14-S15-S2-S2.PDF]
